# Infection of bursal disease virus abrogates the extracellular glycoprotein in the follicular medulla

**DOI:** 10.1016/j.psj.2021.01.023

**Published:** 2021-01-16

**Authors:** Balázs Felföldi, Ildikó Bódi, Krisztina Minkó, Zsófia Benyeda, Nándor Nagy, Attila Magyar, Imre Oláh

**Affiliations:** ∗ Scientific Support and Investigation Unit, Ceva-Phylaxia Co. Ltd., Ceva Animal Health, 1107 Budapest, Hungary; † Department of Anatomy, Histology and Embryology, Faculty of Medicine, Semmelweis University, 1094, Budapest, Hungary; ‡ Prophyl Ltd., 7700 Mohács, Hungary

**Keywords:** bursa of Fabricius, Movat pentachrome staining, extracellular glycoprotein, infectious bursal disease virus infection, bursal secretory dendritic cell transformation

## Abstract

In the medulla of bursal follicle, only the secretory dendritic cell (**BSDC**) is furnished with secretory machinery. The granular discharge of BSDC appears in membrane-bound and solubilized forms. Movat pentachrome staining proves that the solubilized form is a glycoprotein, which fills up the extracellular space of follicular medulla. The glycoprotein contributes to bursal microenvironment and may be attached to the surface of medullary lymphocytes. The secretory granules of BSDC may be fused, resulting in large, irregular dense bodies, which are the first sign of BSDC transformation to macrophage-like cells (**Mal**). To determine the effect of infectious bursal disease virus (**IBDV**) infection on the extracellular glycoprotein and BSDC, SPF chickens were experimentally infected with IBDV. On the surface of BSDC, the secretory substance is in high concentration, which may contribute to primary binding of IBDV to BSDC. The early distribution of IBDV infected cells is in consent with that BSDC. The IBDV infected BSDC rapidly transforms to Mal in which the glycoprotein staining appears. In the dense bodies, the packed virus particles inhibit the virus particles preventing the granular discharge, which may represent the first, early phase of virus replication cycle. The absence of extracellular glycoprotein results in alteration in the medullary microenvironment and subsequently B cell apoptosis. On the surface of medullary B cells, the solubilized secretory substance can be in much lower concentration, which results in secondary binding of IBDV to B cells. In secondary, late phase of virus replication cycle, the virus particles are not packed in electron dense substance which results in cytolytic lymphocytes and presence of virus in extracellular space. The Mal emigrates into the cortex, where induces inflammation, recruiting heterophil granulocyte and monocyte.

## Introduction

Chickens are highly susceptible for infectious bursal disease virus (**IBDV**), but clinical disease most frequently occurs between 3 and 17 wk of age (Fadly and Nazerian, 1983; Vervelde and Davison, 1997). This age-related susceptibility of the bursa of Fabricius may depend on the lymphocyte population of medulla (Ivanyi and Morris, 1976; Brecht, 1980; Kaufer and Weiss, 1980; Okoye and Uzoukwev, 1990; Ramm et al., 1991; Vervelde and Davison, 1997). These researchers suggested that a large number of highly susceptible cells are crucial for clinical IBD (Okoye and Uzoukwev, 1990). Okoye et al. (1992) declared that “healthy bursa is essential for the development of clinical infectious bursa disease (**IBD**)”. This statement is based on embryonically bursectomized and cyclophosphamide-treated chickens, which do not develop clinical IBD because both experiments resulted in B cell depletion. It is generally accepted that the major target of IBDV is the bursal medullary, surface IgM + B cell (Nieper and Müller, 1996; Vervelde and Davison, 1997; Schröder et al., 2001; Withers et al., 2006), but not the “early” lymphoblast (Beug et al., 1981). The IBDV also replicated in the macrophages and granulocytes (Kaufer and Weiss, 1976; Ramm et al., 1991; Khatri et al., 2005). Chicken embryo fibroblast (**CEF**) has been used for propagation of IBDV. Nieper and Müller (1996) proposed that the surface of CEF cells binds a 40-46 kDa protein, which might be a common receptor site for both IBDV serotypes.

The bursal secretory dendritic cell (**BSDC**) produces electron dense, cytoplasmic granules, which may be discharged or fused together (Oláh and Glick, 1978, 1987; Oláh et al., 1979). Exact function of the BSDC is unknown, but we have assumed that the secretory substance contributes to the bursal microenvironment and B cell differentiation and survival (Glick and Oláh, 1984, Glick and Olah, 1993b, Glick and Oláh, 1993a; Oláh and Nagy, 2013). Cyclophosphamide treatment resulted in B cell depletion and large amount of extracellular substance exclusively in the medulla (Oláh and Glick, 1978; Oláh et al., 1979). We suggested that the extracellular substance did come from BSDC, which are embedded in the electron dense extracellular substance. The transmission electron microscope shows that the discharge of granular substance attaches to the external surface of BSDC membrane and appears as membrane-bound substance, which gradually solubilized in the medulla (Oláh and Glick, 1987; Olah et al., 1992a, Olah et al., 1992b; Glick and Olah, 1993b, Glick and Oláh, 1993a). In the medulla, the transmission electron microscope shows a solubilized, fine flocculated substance, which is remarkable around the BSDC.

The aim of this study was to follow the changes of BSDC and their granular content during IBDV infection. The Movat pentachrome staining (Bancroft and Gamble, 2001) provides evidence that the solubilized discharge of BSDC is a glycoprotein (**gp**), which fills up the extracellular space of follicular medulla.

## Materials and methods

### Animals

Three-week-old White Leghorn layer-type chickens of SPF status (Charles River) were used in the test. Sex: mixed. A total of 45 chickens were randomly selected into 3 groups of 15 animals: uninfected control, infected with very virulent IBDV, and infected with variant IBDV. The chickens were reared under standard conditions on deep litter, feed (sterilized broiler grower mix) and water (tap water) was available ad libitum, temperature adjusted to 26 to 16°C. The experiment was conducted in compliance with the Ceva-Phylaxia Scientific Committee on Animal Health and Animal Welfare.

### Infection

Infectious bursal disease virus infection was performed, using very virulent field isolate (D407/02/04/TR) and variant Delaware-E strain. The applied IBDV virus strains were characterized by sequence analysis. The bursa of Fabricius of each animal was cut in pieces collected for multiple morphological studies. The virus suspension was applied via per os at dose of 3.0 lgEID_50_/chicken (diluted in 0.2 mL sterile PBS) Tissue samples were collected at 36 h, 2, 4, 5, and 7 d after infection. The chickens were humanely euthanized by CO_2_ inhalation.

### Antibodies

Anti-IBDV monoclonal antibody (clone: 5A10) specific for VP2 protein was received from Ceva-Phylaxia Hungary. The B cell–specific anti-chB6 (clone: BoA1) antibody was generated in our laboratory (Igyártó et al., 2008).

### Immunocytochemistry

Tissue samples were embedded in liver tissue and snap-frozen in liquid nitrogen. The blocks were stored in a capped 50 mL tube at −80°C until cryostat sectioning. The 10 μm cryostat sections were fixed in cold acetone for 10 min and rehydrated in PBS. The sections were incubated with primary antibodies (5A10 1:500; BoA1 1:10) for 40 min at room temperature. After washing, isotype-specific biotinylated secondary antibody (Vector Laboratories; dilution 1:200 in PBS) was used. The endogenous peroxidase reaction was blocked by 3% H_2_O_2_ for 10 min. VECTASTAIN Elite ABC complex kit (PK-6100; Vector Laboratories) enhanced the signals of primary antibodies. The binding sites of primary antibodies were detected by 4-chloronaphtol (C8890; Sigma-Aldrich Hungary). For control straining, primary or secondary antibodies were replaced by PBS or occasionally an irrelevant isotype-specific antibody was also used.

### Confocal Microscopy

After primary antibodies (BoA1 and 5A10) sections were incubated with Alexa-conjugated fluorescent secondary antibodies: Alexa Fluor 594 goat anti-mouse IgG (A-32742, 1:200), Alexa Fluor 488 goat anti-rabbit (A-11008, 1:200), all from Thermo Fisher Scientific. (1:200). Section images were recorded with confocal microscopy (Zeiss LSM 780 confocal microscope, equipped with Argon multi-line, 405 nm, 561 nm solid state and 633 nm HeNe lasers).

### Light Microscopy

Glycoprotein demonstration (Bacroft and Gamble, 2002) took place by Russel's modified Movat pentachrome staining.

### Transmission Electron Microscopy

Tissue samples were fixed in phosphate buffered 4% glutaraldehyde at 4°C, overnight. PBS removed the excess fixative and the samples were postfixed in 1% osmium-tetroxide for 2 h The samples were dehydrated in graded ethanol and embedded in a mixture of Araldite epoxy resin (Polysciences, Warrington, PA). Ultrathin sections were contrasted with lead-citrate and uranyl-acetate and analyzed with Jeol Jem 1200EX electron microscope.

## Results

**Figure 1:**
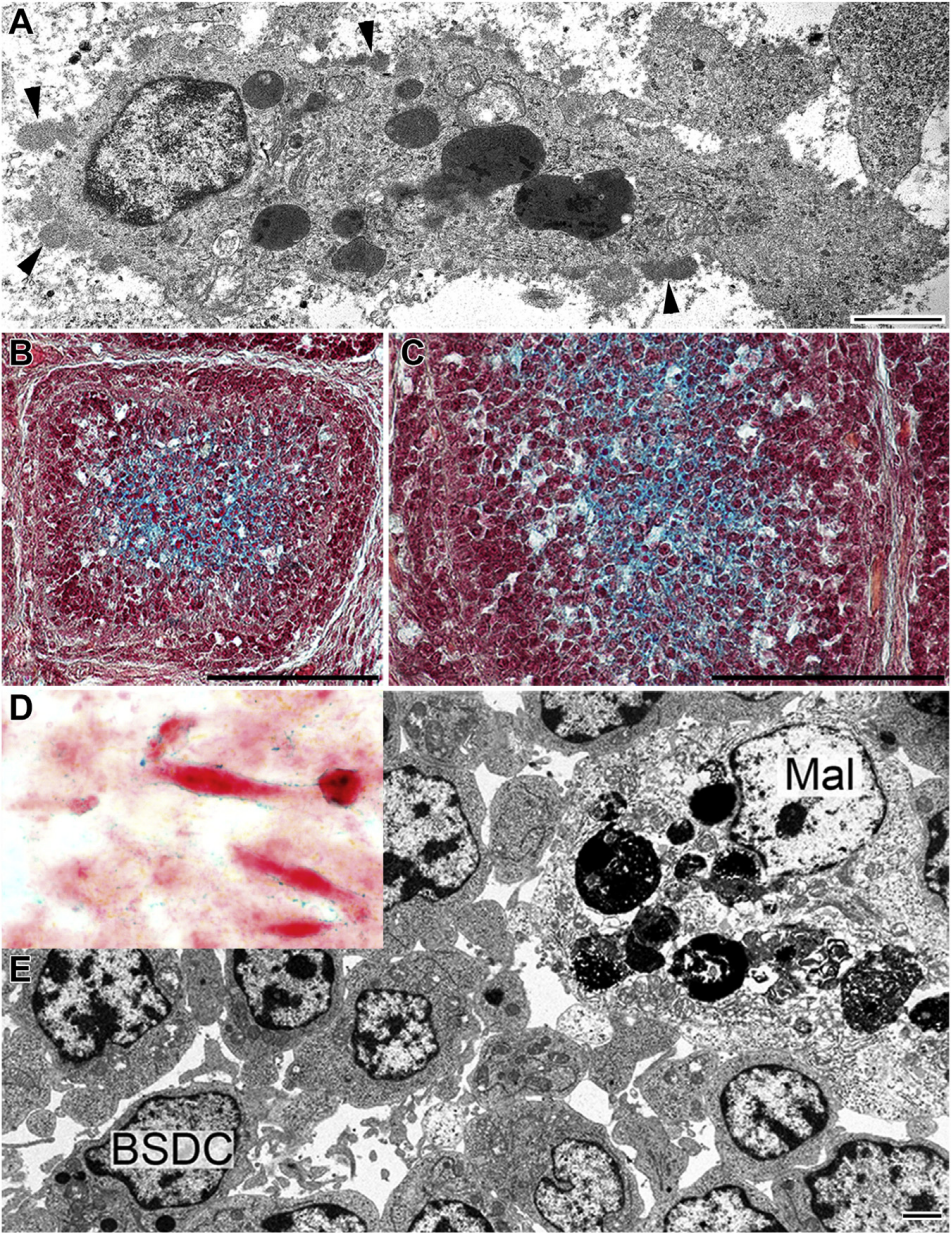
Control birds. (A) Transmission electron micrograph of a BSDC. The size and heterochromatic appearance of nucleus are highly similar to that of a small lymphocyte. The eccentrically located nucleus and the elongated cytoplasm with electron dense granules (some of them fused) give a polarized appearance to BSDC. A spotted, filamentous substance of medium density covers the outer leaflet of plasma membrane. A fine flocculated substance occurs around the BSDC. (B,C) Cross and longitudinal sections of bursal follicles. Movat pentachrome staining shows an extracellular substance in the central portion of follicular medulla. (D) A Movat-positive gp covers the surface of CEF. (E) A young BSDC contains few electron dense granules, but the plasma membrane has not yet shown membrane-bound substance. The bulky cytoplasm of a macrophage-like cell (Mal) contains dense granules and apoptotic cells in different digested states. Abbreviation: BSDC, bursal secretory dendritic cell.

The BSDC locates in the medulla of bursal follicle. The eccentrically located nucleus and the cytoplasmic granules, which occupy one of the cell processes, endow with high polarity to the cell (Figure 1A). The cytoplasmic granules frequently fuse together, forming large, irregular-shaped, electron dense bodies. A spotted filamentous substance covers the surface of the BSDC, which comes from the granular discharge. Around the BSDC, a fine-flocculated substance was found, that is possibly a solubilized form of the surface-bound substance (Figure 1A). Movat pentachrome straining identifies an extracellular substance gp in the medulla of bursal follicles (Figures 1B and 1C) and on the surface of CEF (Figure 1D). This bursa-specific gp may be attached to the surface of medullary lymphocytes (Chechik et al., 1988). In addition to the BSDC, histologically macrophage-like cells (Mal) can be found in the medulla of bursal follicle (Figure 1E).

**Figure 2:**
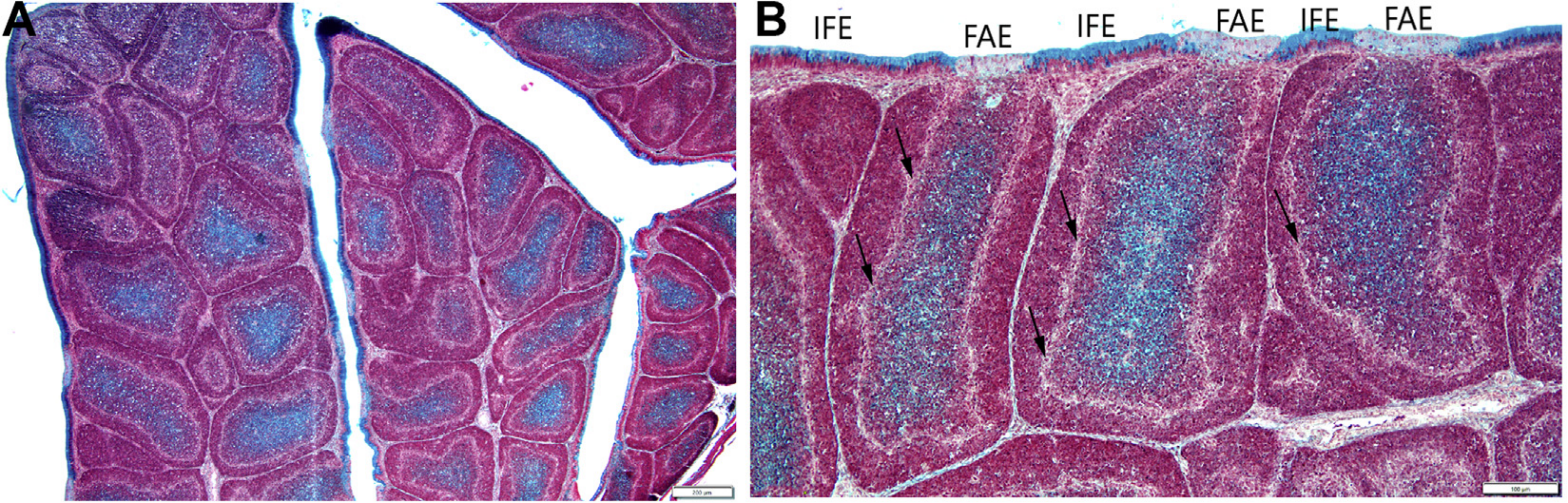
Control birds. (A) Movat stainability varies from follicle to follicle. There are gp devoid of follicles. (B) The IFE shows strong gp staining and the FAE is completely negative. The cortico-medullary epithelial arches appear as a faint line (arrow) that contains histologically lymphoblast-like cells, actually precursors of BSDC. Abbreviations: BSDC, bursal secretory dendritic cell; IFE, interfollcular epithelium.

Movat pentachrome staining recognizes gp in the intracellular mucin of interfollcular epithelium (**IFE**) cells and shows diffuse extracellular staining in the follicular medulla (Figure 2A). In the middle of the medulla, the cells are “floating” in the extracellular fluid gp which is attached to the surface of medullary lymphocytes (Chechik et al., 1988). In the individual follicles, the intensity of the gp staining greatly varies, suggesting different secretory stages of BSDC (Figure 2A) and subsequently different functional phases and immunological responses of individual follicles. Some follicles either are devoid of gp or contain very small amount of gp (Figure 2A). The cells of the follicle-associated epithelium (**FAE**) and the cortex are free from gp (Figure 2B).

**Figure 3:**
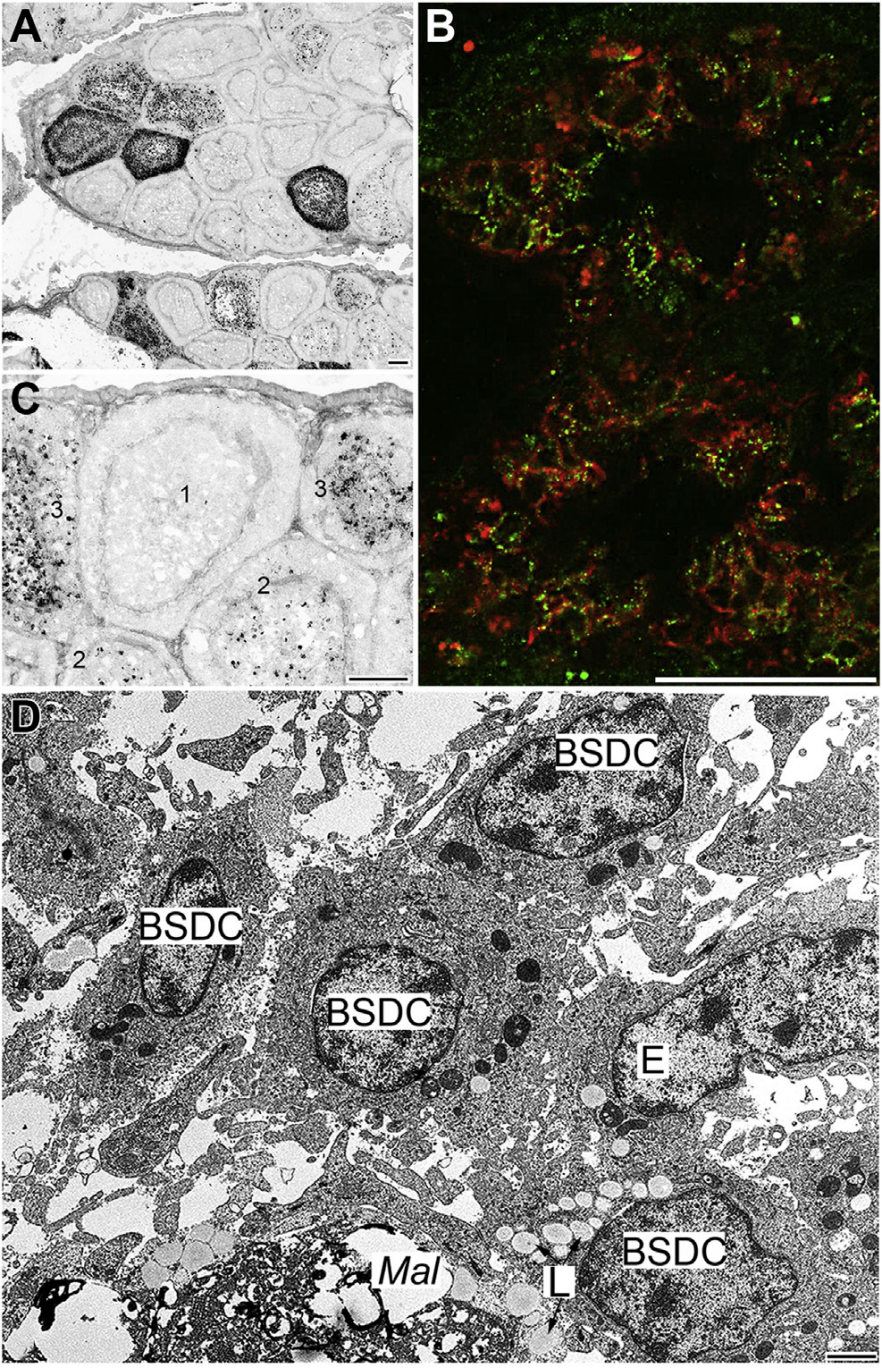
(A) Two days post-infection. The immunocytochemistry of anti-IBDV staining shows highly heterogeneous follicles. (B) Next to the IBDV-negative follicle 1), medulla is moderately infected 2); few IBDV positive cells already appear in the cortex 3). (C) Four days post-infection. Double staining of B cell (red) and IBDV (green). Majority of virus-containing cells are not double stained, Mal (green). Few Bu-1 positive B cells show the presence of IBDV (yellow color). (D) Four days post-infection. In addition to lymphocyte depletion, increased number of young BSDC emerged. The cells are not elongated and few cytoplasmic granules are present, but spotted substance on the cell membrane is absent. In these BSDC lipid droplets (L) may be accumulated in the cytoplasm without phagocytic materials. Abbreviations: BSDC, bursal secretory dendritic cell; IBDV, infectious bursal disease virus.

**Figure 4:**
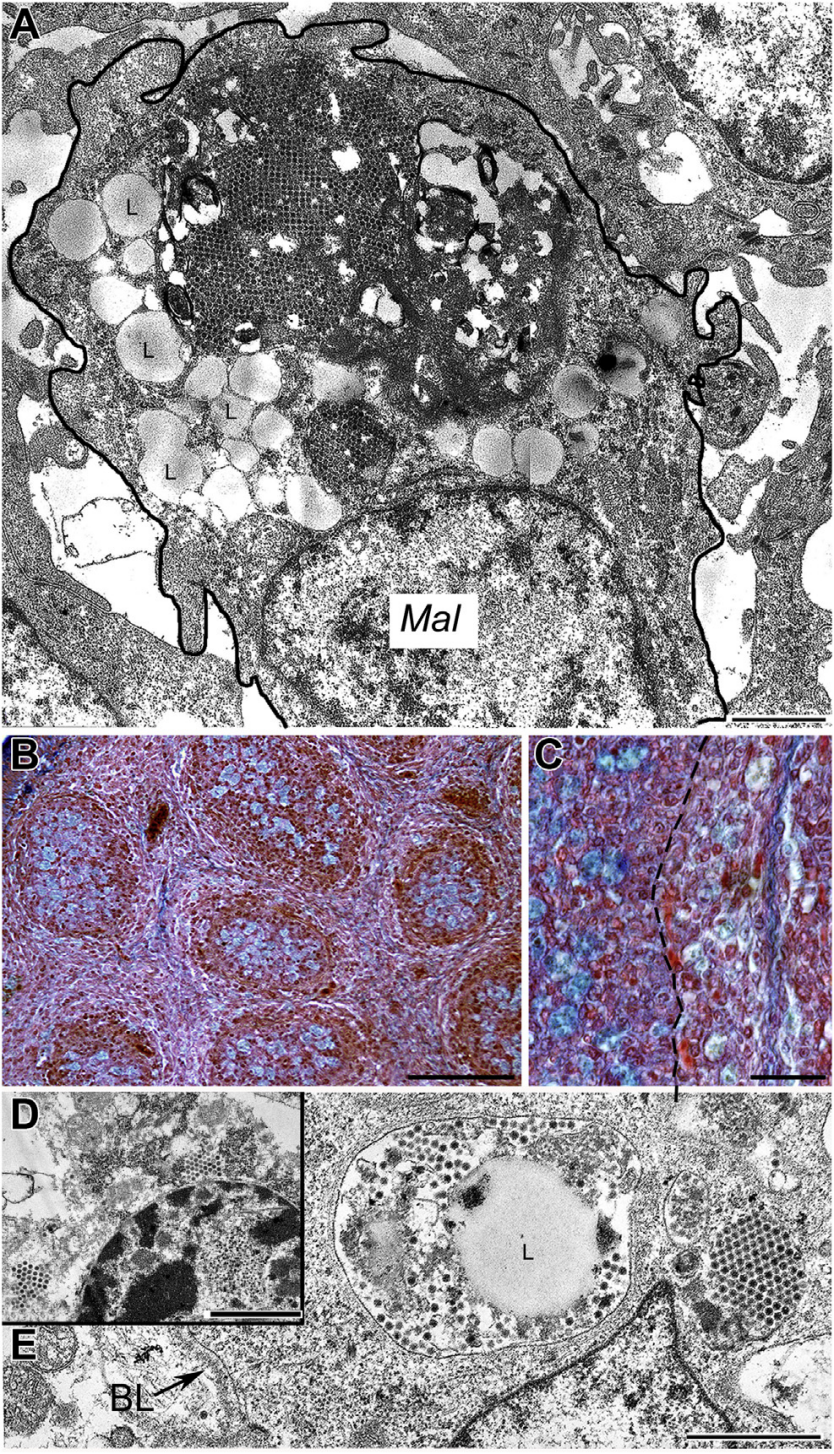
(A) Four days post-infection. The infected cell (Mal) contains giant dense body, which shows lysis, crystalline arrangements of virus particles and lipid droplets (L). (B) Five days post-infection. Movat positive staining appears in the Mal. Extracellular glycoprotein staining ceased. (C) Five days post-infection. In other bird, the Mal emigrated from the medulla. Higher magnification shows faded Movat positive cells (Mal) in the cortex. Dashed line shows the CM border. (D) Two groups of viruses in a lymphocyte, which shows cytolysis. (E) In a CM epithelial reticular cell, virus particles are free in the cytoplasm and scattered around a lipid droplet (L) in a membrane bound vacuole. Abbreviations: BL, basal lamina; CM, corticomedullary.

**Figure 5:**
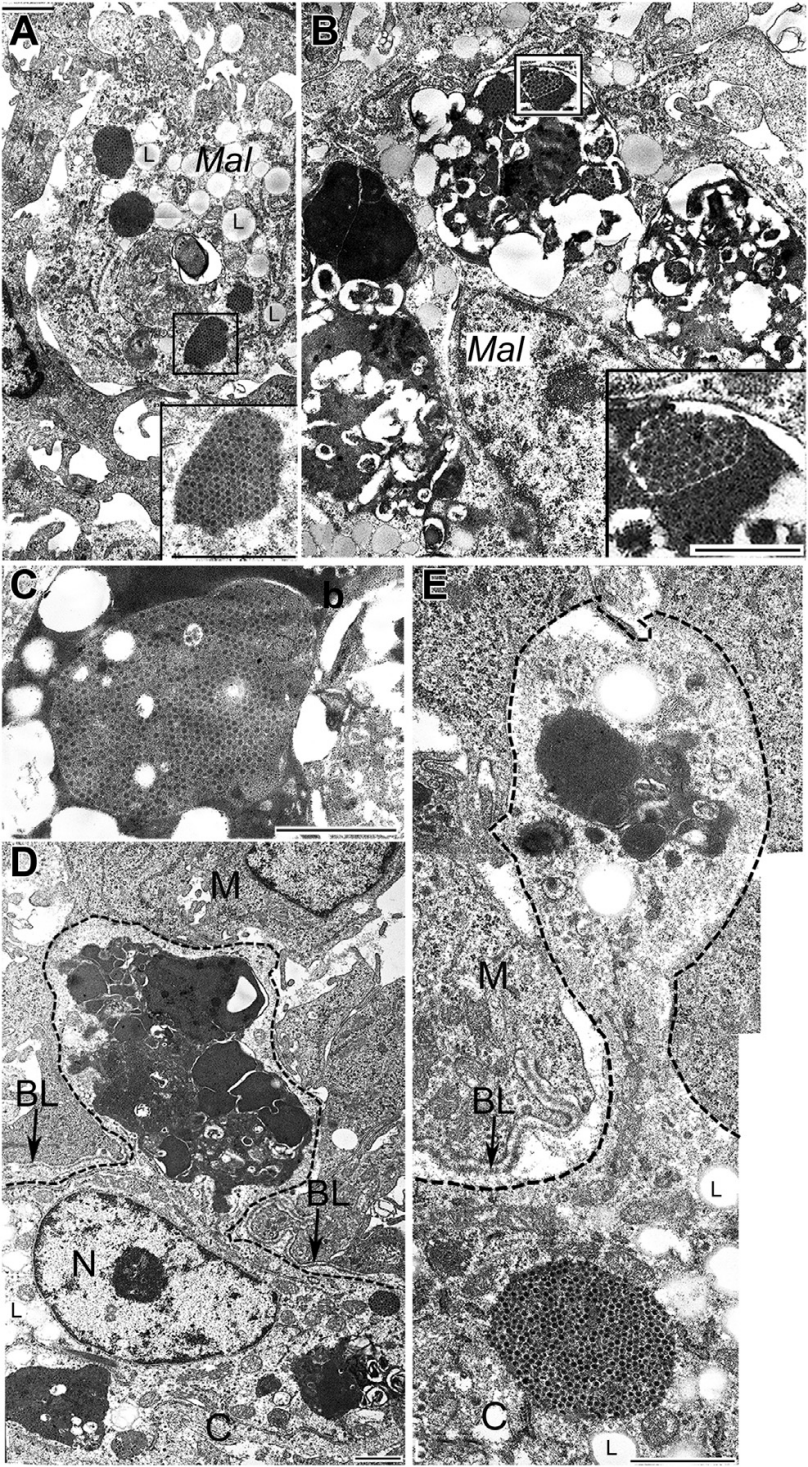
(A) Electron micrograph of a BSDC/Mal. One of the granules contains crystalline arrangement of viruses. Lipid droplets (L) accumulate in the transforming BSDC (compare with Figure 3D). (B) Electron micrograph of a Mal. Four, large dense bodies show breakdown. In one of the dense bodies (outlined) virus particles still can be recognized. Inset: (C) The virus particles are getting to be blurry in a large dense body of Mal. (D) Migration of a Mal through the CM border (BL). The dashed line shows the surface membrane of a Mal. The nucleus (N) of the Mal is in the cortex (C), while part of the cytoplasm with one huge dense body is still in the medulla (M). In a dense granule virus particles are packed, but do not form crystalline arrangement. The dashed line follows the cell membrane of the migrating Mal. Abbreviations: BSDC, bursal secretory dendritic cell; BL, basal lamina; C, cortex; M, medulla; CM, corticomedullary.

At 2 d post-infection (**dpi**), anti-IBDV straining shows highly heterogeneous follicles, from IBDV negative up to the heavily infected ones (Figure 3A). The first IBDV positive cells appear in the medulla (Figure 3B) and their topographical pattern corresponds to that of BSDC. By 4 dpi, many immature BSDC appear following the severe B cell depletion (Figure 3D). Colocalization of B cell and anti-IBDV signals rarely occurs (Figure 3C), suggesting that most virus-containing cells are not B cells. The virus-containing lymphocytes show membrane disintegration (Figure 4D). The virus loaded cells Mal of 20-25 μm size. In the Mal, the virus particles are packed in a high electron dense substance of fused BSDC granules. Besides the huge, virus-containing dense bodies, many lipid droplets are also characteristic structures of transitory forms of BSDC to Mal (Figure 3, Figure 4D, 4A and 5A). The packed virus particles gradually become blurry and their shape fades (Figures 5A-5C). By 5 dpi, the follicles are shrunken and Movat-positive gp disappears from the intercellular space of medulla, but gp staining emerged in the Mal (Figures 4B and 4C). The Mal, loaded with large, electron dense, virus-containing bodies migrates into the cortex (Figures 5D and 5E). The appearance of faint Movat-positive cell in the cortex (Figure 4C) supports the migration of Mal into the cortex.

Occasionally, virus particles enter the medullary epithelial reticular cells, but these intraepithelial virus particles are either free in the cytoplasm or in a vacuole. In the epithelial cells and lymphocyte, the virus particles are not packed in a high electron dense substance, like in case of Mal (Figures 4D and 4E).

## Discussion

### Source of Glycoprotein

In the bursal follicular medulla, the BSDC is the only secretory type cell that we know. The BSDC produces different-sized, electron-dense granules, which either discharge or fuse together, forming large, irregular dense bodies. The discharged granular substance appears as a spotted material on the BSDC membrane. The spotted material may be detached from the cell membrane and appears in the medulla as a fine-flocculated, solubilized substance. Movat pentachrome staining shows that a solubilized gp fills up the extracellular space of the follicular medulla. This gp may be provided a unique microenvironment for medullary B cells because it attaches to the surface of medullary lymphocytes (Chechnik et al., 1988).

### BSDC Transforms to Macrophage-like Cell

Histological findings (light and transmission electron microscopic) are in conflict with the immunocytochemistry. In the medulla histologically, Mal cells, can be identified, but there is no a specific monocyte-macrophage marker recognizing medullary macrophages (Mast et al., 1998; Jeurissen et al., 1992). Recently, Hu et al. (2019) described a novel antibody, which recognize the avian T cell immunoglobulin and mucin domain–containing 4 (**TIM4**) antigen specific for of subpopulations of chicken mononuclear phagocytes and a subset of bursal B cells. Between BSDC and medullary macrophage, we have observed transitory forms, which contain phagocytosed, apoptotic lymphocytes, and spotted extracellular substance on the cell membrane (Oláh et al., 2014). These observations show that the medullary macrophage represents a unique macrophage population, that we call Mal. Possibly, the BSDC physiologically transforms to phagocytic, Mal which is capable of leaving the medulla either via follicle-associated epithelium (Houssaint and Hallet, 1986), or by crossing the corticomedullary border (Oláh et al., 2001). Possibly the IBDV infection accelerates the transformation of BSDC to Mal because the decreased number of functioning BSDC could be a feedback to the BSDC precursors—lodging in the corticomedullary epithelial arches—to differentiate mature cells. At 4 dpi, this feedback mechanism may be justified by the increased number of BSDC. It is worthy to mention that in the epithelial cells and lymphocytes, the virus particles are not packed in a dense substance, like in the Mal. These viruses are not blurred—like in the Mal—and their elimination may be slower.

In control birds, the BSDC granules do not show Movat staining; gp is present extracellularly. However, after infection, the gp staining appears intracellularly, in the Mal. These findings suggest that the infection alters the function of BSDC. Possibly, the discharge of the granules or virus packaging, results in conformational changes in the granular content, results in appearance of Movat stainability appears either in the extracellular space or in the Mal. We assume that in chicken, the gp contributes in a paracrine manner to B cell survival because gp binds to the medullary lymphocytes (Chechik et al., 1988). Infectious bursal disease virus replication cycle can be divided for 2 stages: 1) an early phase, in which the virus particles are released from metabolically active cells by nonlytic egress mechanism, and 2) a late phase, which is associated with pathogenic effect or cell death (Mendez et al., 2017). Our results, that BSDC transformation to Mal is accelerated by IBDV, seem to support the first phase of replication and release of virion from Mal, while during the second phase of replication, the medullary lymphocytes are infected and this phase is associated with lytic egress of virus particles. The concentration of membrane-bound and solubilized forms of the BSDC secretory product may explain the primary and secondary binding of IBDV. The high concentration of gp on the surface of BSDC may result in primary binding of IBDV and the lower concentration on the surface of medullary B lymphocytes could be responsible for secondary binding. The CEF is capable of binding large amount of virus particles from both serotypes. In CEF culture, there is 40-46 kDa cell surface protein, which functions as early step of IBDV binding (Nieper and Müller, 1996). Movat staining proves that surface of cultured CEF is covered by gp. Migration of Mal into the cortex results in inflammation and heterophil accumulation in the cortex. In *in vitro* studies, the time course of virus replication (Mendez et al., 2017) is closely harmonized by that of our *in vivo* observations.

The first IBDV positive cells appear in the medulla (Schröder et al., 2001) and their topographical pattern is similar to that of BSDC. In the infected birds, the irregular-shaped dense bodies (coming from the fused granules) pack in the virus particles, which stop the granular discharge from BSDC resulting in absence of extracellular gp. Changes in the bursal microenvironment may initiate B cell apoptosis. Transmission microscope shows virus particles in “normal” and cytolytic, non-apoptotic lymphocytes. This finding conflicts with earlier reports of Lam (1997) and Ojeda at al. (1997), which proposed that virus infection directly resulted in B cell apoptosis. In double-stained sections, B cells rarely colocalize with anti-IBDV signal suggesting that the major target of IBDV is not lymphocyte.

The stainability of the extracellular mass of the medullary gp varies from follicle to follicle, which may be contributed to the functional phase of the individual follicles. The gp is a product of BSDC (Olah and Glick, 1978; Olah et al., 1979); therefore, the functional phase of a follicle may depend on the BSDC discharge. There are follicles that form to be devoid of gp, which suggests that the granular discharge from BSDC(s) is synchronized inside the follicles. If this is true, the BSDC is a regulatory cell of the follicular medulla, and it may explain the presence of IBDV-free follicles among the heavily infected ones.

The production and location of Movat-positive gp shows some similarities to those of B cell activating factor (**BAFF**). In mammals, dendritic cells, macrophages, and myeloid cells produce BAFF, which in a paracrine manner contributes to proliferation and survival of mature B cells (Moore et al., 1999; Nardelli et al., 2001; Mackay et al., 2003). However, in chicken, the bursal B cells are the major source of BAFF (Koskela et al., 2004; Schneider et al., 2004; Kothlow et al., 2007), and in an autocrine manner promotes the proliferation and survival of B cells (Schneider et al., 1999; Schneider et al., 2004). In situ hybridization signal of BAFF is restricted to the follicular medulla (Kothlow et al., 2007). However, spleen does not have detectable level of BAFF transcript; therefore, mature B cell could not be the source of BAFF. Schneider et al. (2004) assumed that BAFF is synthetized by bursal stromal cells or immature B cells.

### What Could Be the Extracellular Glycoprotein in the Follicular Medulla?

Exact role of the gp is unknown. Immunoglobulins (Ig) are gp(s). It was reported that Ig(s) are regularly present in the extracellular space of follicular medulla and their appearance is related to the stage of the bursa development; namely, during the first days after hatching, considerable changes occur in the Ig-containing cells of lymphoepithelial follicles (Zaccheo et al., 1969). (Bursal follicles are histologically lymphoepithelial follicles). Our observation, on the immature BSDC, confirms the abovementioned statement. Around hatching the round-shaped, immature BSDC binds possibly maternal IgY, before its differentiation is not completed. The differentiated BSDC is highly polarized and granular discharge begins (Oláh and Glick, 1995). In control birds, the gp is extracellularly, whereas during IBDV infection, the Movat staining shows gp in the Mal. These findings do not support that the extracellular gp is being Ig(s).

Macrophages and other types of cells can secrete thrombospondin (**TSP**), which is an adhesive gp and implicates in many embryo, morphogenetic, and pathogenic events (Savill et al., 1993). The transforming of BSDC to Mal may secrete TSP, which may contribute to the ligand mediating uptake of apoptotic and cytolytic B cells. In control birds, the Movat stainability of extracellular gp changes from follicle to follicle, which suggests that the gp rather plays a role in physiological or immunological functions of medulla than pathological ones.

The **IFE** produces a Movat-positive substance, mucin, which contains gp. However, the Movat-negative **FAE** (Bódi et al., 2019) is in topographical connection with the medulla; therefore, the presence of IFE-produced mucin in the medulla is highly questionable. The serious IBDV infection results in B cell depletion and the FAE gives away; subsequently the follicular medulla may be transformed to an IFE-lined vesicle, which is filled up by the Movat-positive substance, mucin. In less serious condition, the gp is in the Mal.

Immunoglobulins, TSP, and mucin are excluded; therefore, the Movat-stained medullary gp can be a bursa-specific substance.
